# Nb-Based Zeolites: Efficient bi-Functional Catalysts for the One-Pot Synthesis of Succinic Acid from Glucose

**DOI:** 10.3390/molecules22122218

**Published:** 2017-12-14

**Authors:** Magdi El Fergani, Natalia Candu, Simona M. Coman, Vasile I. Parvulescu

**Affiliations:** Department of Organic Chemistry, Biochemistry and Catalysis, Faculty of Chemistry, University of Bucharest, Bdul Regina Elisabeta 4-12, Bucharest 030016, Romania; magdi.belkassim@yahoo.com (M.E.F.); natalia.candu@chimie.unibuc.ro (N.C.)

**Keywords:** Beta-zeolite, niobium, dealumination, post-synthesis insertion, glucose, oxidation, succinic acid

## Abstract

The one-pot production of succinic acid from glucose was investigated in pure hot water as solvent using Nb (0.02 and 0.05 moles%)-Beta zeolites obtained by a post-synthesis methodology. Structurally, they are comprised of residual framework Al-acid sites, extra-framework isolated Nb (V) and Nb_2_O_5_ pore-encapsulated clusters. The Nb-modified Beta-zeolites acted as bi-functional catalysts in which glucose is dehydrated to levulinic acid (LA) which, further, suffers an oxidation process to succinic acid (SA). After the optimization of the reaction conditions, that is, at 180 °C, 18 bar O_2_, and 12 h reaction time, the oxidation of glucose occurred with a selectivity to succinic acid as high as 84% for a total conversion.

## 1. Introduction

The use of the biomass as feedstock ensures the sustainability of resources and, on this basis, it is of a high current interest for the chemical industry [1,2,3,4,5]. Accordingly, the conversion of glucose into monomers emerged as very important. Therefore it is necessary to make it available in large quantities via either chemical or enzymatic hydrolysis of both cellulose and hemicelluloses [6]. However, to answer the complexity and diverse reactivity of the various biomass substrates, it is necessary to develop novel catalytic systems able to afford with high reaction rates the required conversions and selectivities of the feedstocks [7,8].

The wet oxidation (WO) of glucose can provide, in principle, a very appealing green route for the synthesis of platform molecules usable for the monomers preparation, where the solvent is water and molecular oxygen is the only used oxidation reagent. However, in the absence of a catalyst, the WO of glucose is a very unselective process [9,10,11,12].

In this context, we reported not long ago that ruthenium-amine (i.e., *n*-butylamine) complexes improve the selectivity of glucose WO by favoring the formation of certain reactive oxygen species and promoting some pathways that would not take place in its absence. The catalytic wet oxidation (CWO) takes place, in this case, with the total conversion of glucose and the selective formation of succinic acid (SA, selectivity values above 85%), highly used in the preparation of polyesters and polyamides [13]. The beneficial influence of *n*-butylamine was explained by several effects, such as: (i) it favors the desorption of the generated carboxylic acids from the Ru(III) species; (ii) it increases the stability of the Ru nanoparticles (NPs), and; (iii) it stabilizes the catalytically active species, namely, Ru(IV) species.

An important issue to ensure the economic attractiveness of converting glucose directly to SA by oxygen, in aqueous medium, is to improve the stability of the Ru(III) catalyst, particularly to find alternatives for the continuous addition of an excess of amine. Therefore, a step forward represented the development of ruthenium oxyhydroxide nanoparticles supported on N-doped graphene catalysts. They are able to convert glucose to SA (selectivity of 87%) in the absence of an excess of amine [14].

Although the use of ruthenium catalysts is considered as a breakthrough in the valorization of glucose, there are still several drawbacks associated to its use, and the most important is connected to its critical element status [15]. Its utilization in a nanoparticulate form may offer an efficient use potential, but the metal-free catalytic alternatives or the use of the non-noble metals-based catalysts is more challenging to improve the desired sustainability. In this context, we shown that N-containing graphenes obtained either by simultaneous amination and reduction of graphene oxide or by pyrolysis of chitosan under inert atmosphere may act as metal-free catalysts for the selective WO of glucose to SA [16]. However, the process is less efficient compared to corresponding Ru-based graphene catalysts [14]: at 160 °C, under 18 atm O_2_ and after 20 h the selectivities to SA were in the range of 60–68% at a complete glucose conversion.

Due to their remarkably high acidity (H_o_ = −8.2), also preserved in water, niobia-based catalysts were extensively investigated by different research groups, in various transformations of biomass derivatives [17,18,19,20,21,22]. The enhancement of both the hydrothermal stability and acidity in aqueous phase reactions through the deposition of niobia on oxide supports (e.g., silica, titania, alumina) is well documented in the literature [23]. Moreover, in the presence of Nb-based carbon nanotubes (CN) catalysts, levulinic acid and glucose were successfully oxidized to SA, with selectivities of 98–99% and 80%, respectively, for a total conversion of the raw material at 180 °C, 10 bar O_2_, and after 6 h [21]. However, the catalytic performances, expressed in terms of succinic acid yields, were directly correlated to the nature of the catalytic sites which, in turn, has been dictated by the niobia content and the preparation route. Therefore, the lower the niobia content in the catalyst, the higher the selectivity to SA.

All the above described catalytic materials require quite laborious and time spending preparation methodologies. In this respect, a simpler and faster alternative might afford an easier transfer of the already known structural and chemical properties of the catalysts utilized in the oil-based industry [24,25] to the new biomass-based industry. Thus, for the particular case of the production of succinic acid through the one-pot transformation of glucose, synthetic zeolites are potential candidates, while nanoparticles of niobium can offer a viable alternative to ruthenium since the use of ruthenium as catalytic active specie is inconvenient from the elemental sustainability point of view. To obtain this, the low stability of zeolites in hot water should be improved through a heteroatom insertion. In this respect, the generation of heteroatomic Lewis acid- [26,27] or bifunctional-zeolite catalysts [28] for the production of the platform molecules is an expanding field.

Based on this state of the art, in this work we focused our interest on a post-synthesis strategy for the production of highly dispersed Nb species (0.02–0.05 moles %) into the BEA-zeolite matrix. This strategy supposes a successive zeolite dealumination followed by its impregnation with Nb etoxide. The new catalysts demonstrated a high catalytic efficiency for the direct synthesis of succinic acid from glucose and a very good stability against water. The reactions carried out with these catalysts in water, as solvent, and molecular oxygen, as solely oxidation reagent, afforded the direct oxidation of glucose to succinic acid with selectivities up to 84% for a total conversion of glucose.

## 2. Results and Discussion

### 2.1. Catalysts Characterisation

**Figure 1:**
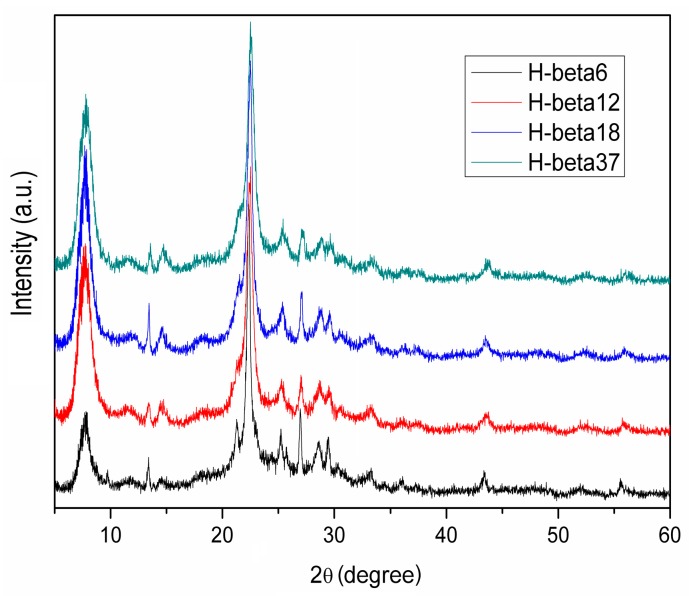
X-ray diffraction patterns of H-β samples.

The X-ray diffraction patterns of the studied H-β samples are shown in Figure 1. The characteristic sharp and intensive reflections of H-β zeolites, at 2θ values of 7.6°, 21.2° and 22.4°, were consistent with the typical diffraction lines of the referenced Beta zeolite, indicating a well-developed crystalline structure of Beta topology, formed by the intergrowth of polymorphs A and B [29].

The crystallinity of the samples was defined on the basis of the three characteristics diffraction lines of Beta zeolite at Bragg angles of 7.5°, 21.3° and 22.4°, and it was calculated by using Equation (1) [30], considering as reference the Beta zeolite sample with the highest intensity of the diffraction of these three lines.

(1) %Crystallinity = ∑i=13Intensityi[∑i=13Intensityi]Ref × 100

Beta zeolites with a higher Si/Al ratio are characterized by a higher crystallinity (Table 1), that is, in a good concordance with previous studies [30,31].

Sharper and more intense reflections of H-β6 (Figure 1) indicate highly regular structures and slightly larger crystals that could be associated with the higher Al content (Si/Al = 6 for H-β6 versus Si/Al = 12 for H-β12). Moreover, all the reflection lines of H-β6 sample were shifted to lower 2θ values than for H-β12, indicating an increase in the unit cell size that could be associated to an increased concentration of longer Al-O bonds compared to Si-O bonds.

In contrast to other zeolites, the structure of the hydrated Beta zeolite shows a reversible change in the coordination of the framework Al atoms. Depending on the counter-ion it may balance the framework into a negative charge. Thus, it is generally accepted that in the hydrated protonic form of the Beta zeolite part of the framework Al atoms, with tetrahedral coordination in Na form, changes the coordination to octahedral [32].

**Figure 2:**
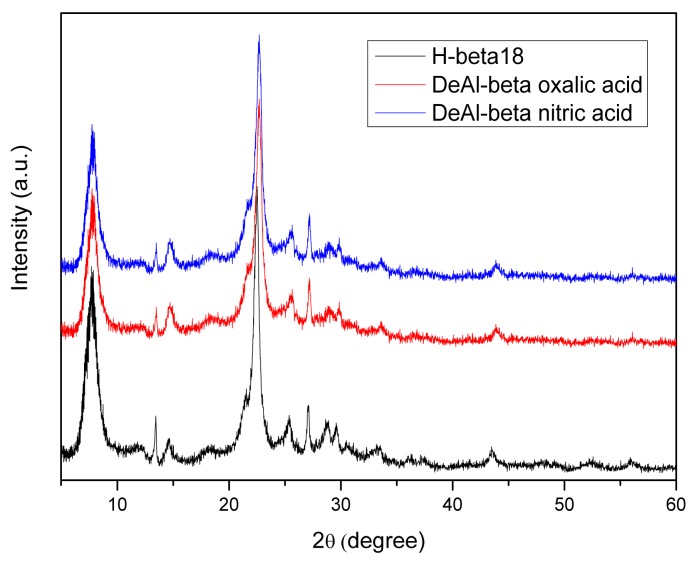
X-ray diffraction patterns of H-β18 and the corresponding dealuminated samples (DeAl-Beta). Treatment in nitric (blue) and oxalic acid (red), respectively.

On the other hand, in an aggressive environment such as HNO_3_ acid, a severe structural collapse of the Beta zeolite may occur. Under these conditions the produced mesoporosity is not useful because the resulted pores collapse and, therefore, the direct connection with the external surface of the zeolite particles is hindered. However, in the present case, the presence and the intensity of the typical diffraction lines of the Beta zeolites at 2θ of 7.6°, 21.2° and 22.5° (Figure 2) certify that the dealumination conditions utilized in this work affected only in part the crystallinity of the parent zeolite, but no obvious broad peak due to the presence of an amorphous (silica-alumina) phase has been observed.

The X-ray diffraction (XRD) pattern of the DeAl-Beta sample prepared through the dealumination with oxalic acid (Figure 2, red pattern) shows a change in the position of the narrow line from 2θ 22.48° to 22.75°, corresponding to a decrease of the d_302_ spacing from 3.952 Å (H-β18) to 3.906 Å (DeAl-Beta). The dealumination with nitric acid changed the position of this diffraction line to 22.67° that correspond to a d_302_ spacing of 3.917 Å. These shifts, in fact, confirm a larger contraction of the zeolite matrix [33] by the treatment with oxalic acid. In conclusion, it emerges that the oxalic acid is more effective for the extraction of lattice aluminum than nitric acid. The extraction occurred without a collapse of the zeolite framework structure. This behavior should be associated with the dual nature of the oxalic acid. In relation to the framework aluminum it may act both as a hydrolyzing and a chelating agent. The dealumination process takes place via the formation of trioxalato aluminum complexes with a high complexation constant (logβ3 = 15.1) [34].

**Figure 3:**
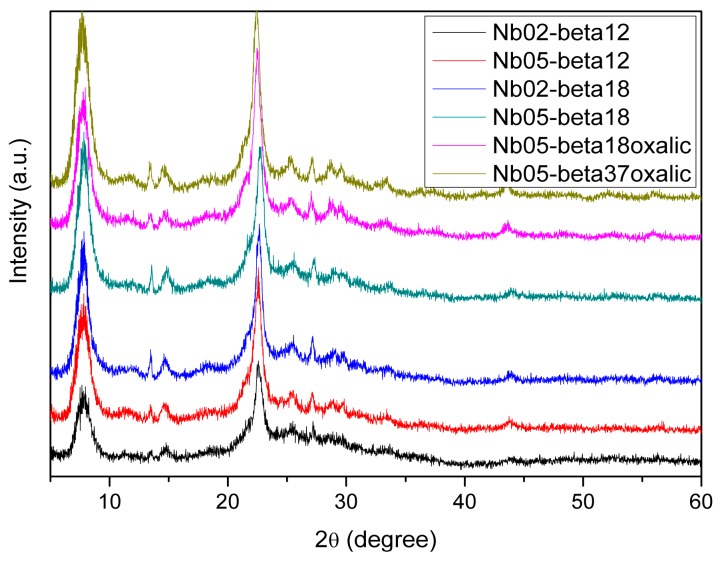
X-ray diffraction patterns of Nb-β samples.

Further, the treatment of the DeAl-beta samples with a niobium salt led to an increase in the d_302_ spacing. Figure 3 presents the XRD patterns of the Nb-treated samples, while the changes in the position of the diffraction line from 2θ 22.48° and the corresponding d_302_ spacing are listed in Table 2.

As Table 2 shows, upon the incorporation of Nb into the DeAl-β samples, the increase of d_302_ spacing compared to the DeAl-β reference depends on the dealumination route and loading of niobium. Finally, this corresponds to different expansions of the Nb-β zeolite matrix. However, the size of the niobium ions and the bond length of Nb-O (1.89 Å for tetracoordinated Nb (V) species in Beta zeolite) compared to that of Si-O (typically 1.60–1.65 Å in zeolites) [35] do not support an insertion of Nb in the tetrahedral positions of the Beta matrix. The d_302_ spacing values measured in this work are too small to sustain such an effect. Larger d_302_ spacing values were measured for the samples dealuminated with oxalic acid (Nb(0.05)-β18O and Nb(0.05)-β37O, Table 2, entries 8 and 10). In these cases the d_302_ spacing values increased from 3.906 Å (DeAl-β18 (oxalic acid)) to 3.936 Å (Nb(0.05)-β18O) and from 3.912 Å (DeAl-β37 (oxalic acid)) to 3.969 Å (Nb(0.05)-β37O) and the shifts are close to those reported by Dzwigaj et al. [36] (i.e., an increase of the d_302_ spacing from 3.912 Å (SiBeta) to 3.954 Å (NbSiBeta, 2θ = 22.45°) for a 0.02 mol % Nb-SiBeta material prepared by the post synthesis methodology.

The inductively coupled plasma optical emission spectrometry (ICP-OES) measurements of the liquid phase separated after the Nb insertion step did not evidence the presence of niobium species suggesting, indeed, an entrapment of all niobia in the Beta zeolite structure. Therefore, it can be stated that for the samples dealuminated with nitric acid the low expansion of the Beta zeolite matrix is most probably the effect of its distortion by the non-framework pore encapsulation of Nb(V) nano-oxides. This process takes place without a real replacement of the tetrahedral aluminum in the zeolite framework. On the contrary, the dealumination with oxalic acid allows the insertion of Nb in the tetrahedral positions of the Beta matrix, at least partially.

**Figure 4:**
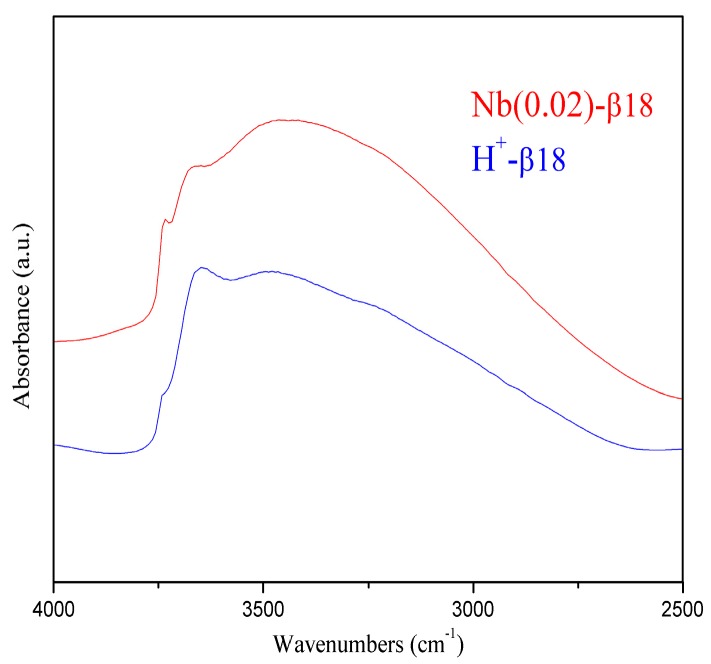
FTIR spectra of H-β18 and Nb(0.02)-β18.

**Figure 5:**
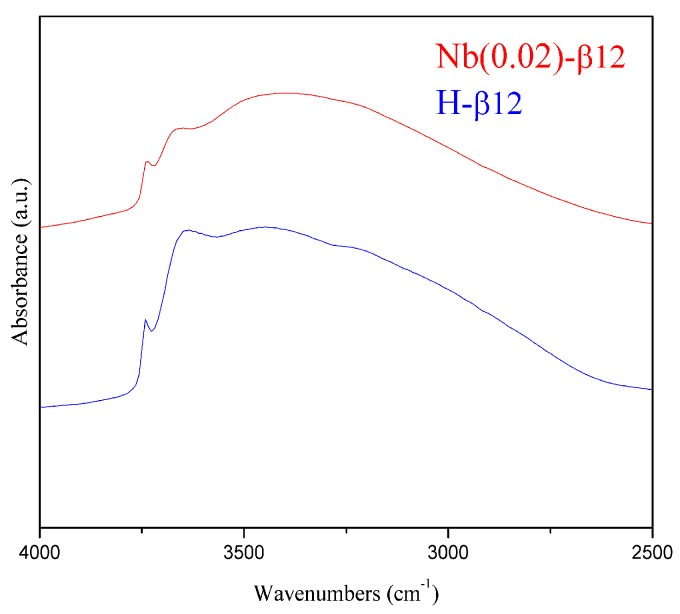
FTIR spectra of H-β12 and Nb(0.02)-β12.

The structural modifications of the zeolite matrix after the insertion of Nb were also evidenced by the FTIR study, confirming the XRD analysis results. In the 4000–2500 cm^−1^ range, the FTIR spectra of all H-β and Nb(0.02)-β samples (Figure 4 and Figure 5) show a broad band assigned to the stretching vibrations of the -OH groups from H_2_O adsorbed on the zeolite surface and/or existing in the zeolite channels (surface silanol groups, Si-OH). The sharp bands from this region can be associated with the asymmetric vibrations of some M-OH isolated groups. The FTIR spectra of H-β18 and H-β12 show typical -OH vibrations in Beta zeolites with bands of non-interacting bridging hydroxyls and the broad band ranging from 3620 to 3200 cm^−1^ of mutually interacting Si(OH)Al groups. The OH stretching modes at wave numbers at 3642 cm^−1^ (H-β12) and 3647 cm^−1^ (H-β18) are due to the Si-O(H)-Al groups, at 3743 cm^−1^ is assigned to terminal silanol (Si-OH) groups in small crystallites and high external surface area materials, while that at 3667 cm^−1^ is attributed by some authors to AlO-H groups of perturbed and extra-framework Al atoms [37].

**Figure 6:**
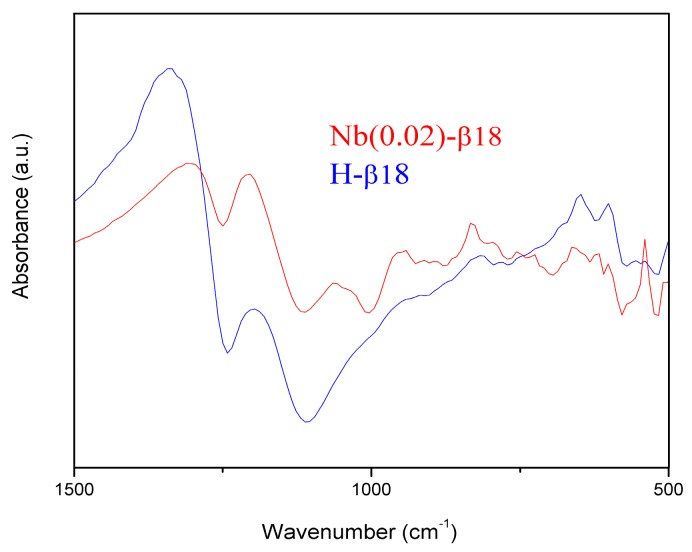
FTIR spectra of H-β18 and Nb(0.02)-β18.

**Figure 7:**
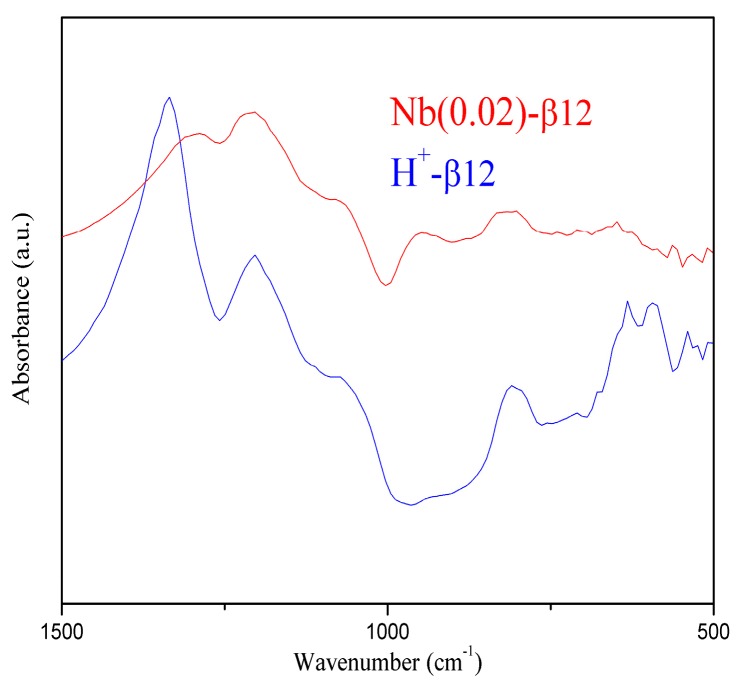
FTIR spectra of H-β12 and Nb(0.02)-β12.

The incorporation of Nb induced a reduction of all the above IR bands’ intensity and new bands centered at 3736 and 3671 cm^−1^ were evidenced, most probably related to the presence of Nb(V)O-H and isolated SiO-H groups. At the same time, no absorption bands at 3555–3602 cm^−1^ were evidenced, confirming the lack of Nb(IV), in accordance with a previous report of Tielens et al. [36]. In 1500–500 cm^−1^ range, corresponding to the framework vibration region, the presence of a new absorption band centered at 952 cm^−1^ has also been evidenced (Figure 6 and Figure 7) for the Nb-based catalyst. According to literature, this band is generally taken as an indication of the metal incorporation into the zeolite framework and based on the reports of Corma et al. [38] it can be attributed to the Si-O-Nb vibrations. However, it is more probable that the presence of such bonds correspond to the extra-framework isolated Nb(V)O-H species. The absence of a band located at 884 cm^−1^ indicates the absence of the niobyl (-Nb=O bonds) species that may also account for the presence of the Nb_2_O_5_ clusters [36].

A sharp peak around 1100 cm^−1^ is usually noticed in zeolites and assigned to the asymmetric stretching of the SiO_4_ tetrahedra. The shift to higher wave numbers is due to the presence of large amounts of other cations, since the Si-O bond distance is shorter than the Al-O one. Indeed, in the case of both the H-β rich-aluminum samples this characteristic band was evidenced at 1197 cm^−1^ (H-β18) and 1202 cm^−1^ (H-β12), respectively (Figure 6 and Figure 7). These bands can be associated with the remnant AlO-H group after the dealumination and Nb insertion, indicating either a partially replacement of the framework Al during the dealumination or the presence of the extra framework AlO_x_(OH) species.

Indeed, it is already well known and documented that during the zeolite framework dealumination the Si-O-Al bonds are hydrolyzed, and tetrahedrally coordinated Al is extracted from the zeolite framework and deposited mostly as octahedrally coordinated mono- and even oligomeric Al-O extra-framework species [39,40]. Moreover, such residual extra-framework aluminum species adds Lewis acidity to the synthesized material [41].

The geometry of Nb is dependent on the synthesis method [36]. Indeed, the present procedure led to extra framework Nb(V)O-H species, while in zeolites prepared through a direct synthesis procedure tetrahedral species presenting a niobyl bond (-Nb=O) have been identified [38]. However, in the case of the dealuminated samples with oxalic acid the presence of penta-coordinated framework Nb(V)O-H species should not be excluded.

### 2.2. Catalytic Behaviour

The aim of this study was to evaluate the catalytic activity of Nb-based zeolites samples for the selective CWO of glucose to SA. It is well known that the product distribution of WO remarkably depends on the experimental conditions and the reaction time as consequence of the instability of the primary products, their evolution over the time and the simultaneous concurrency of different reaction pathways. On the other hand, the catalytic performances are highly influenced by different catalytic system features as reaction conditions, catalytic active phase characteristics and the support nature. Therefore, it has been investigated the influence of the oxygen pressure, reaction temperature and reaction time upon the reaction products distribution.

**Figure 8:**
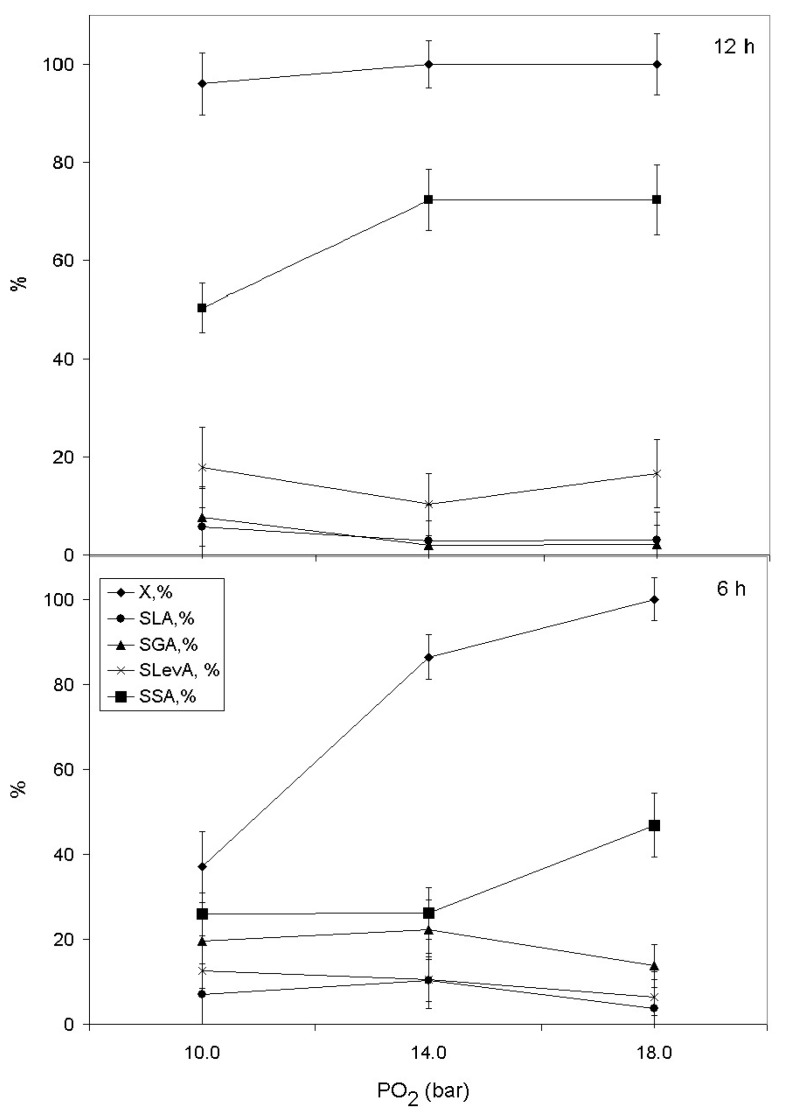
The influence of the oxygen pressure and reaction time upon the reaction product distribution (Reaction conditions: 90 mg glucose; 50 mg Nb(0.05)-β18 catalyst; 10 mL water, 180 °C. LA—lactic acid; GA—glycolic acid, LevA—levulinic acid; SA—succinic acid). Note that the values from graph are averaged ± %RSD (*n* = 3).

Figure 8 presents the influence of the oxygen pressure and reaction time upon the distribution of the most important products, in the presence of the Nb(0.05)-β18 catalyst. As Figure 8 shows, both the glucose conversion and selectivity to succinic acid increased with the oxygen pressure and reaction time. Practically, a total conversion of glucose was reached after 6 h, with a selectivity to succinic acid of 46.8%, at 18 bar O_2_. However, the selectivity to succinic acid continued to increase in time and, after 12 h, reached 72.3%.

Lower loadings of niobium (i.e., 0.02 mol %) and reaction temperatures (i.e., 160 °C) led to lower selectivities to succinic acid (Table 3). Interesting enough, the selectivity to succinic acid is favored by the presence of catalysts with higher Si/Al ratios. In accordance, the highest selectivity to SA (83.6%) for a total conversion of glucose was obtained in the presence of Nb(0.05)-β37.5, dealuminated with nitric acid (Table 3). The corresponding Nb(0.05)-β37.5O sample, produced in the presence of oxalic acid, led to a selectivity of succinic acid of only 70% for a total conversion of glucose.

It is likely that lactic and glycolic acids are formed via the already proposed mechanism of the glucose degradation [20]. On bifunctional catalysts, levulinic acid (LevA) can be also produced from glucose, usually in two steps involving the glucose isomerization to fructose on Lewis acid centers, followed by the fructose dehydration to LevA through 5-hydroxymethylfurfural (HMF) intermediate on Brønsted acid centers [42,43]. However, the dehydration of glucose to levulinic acid through a levoglucosane intermediate is also possible on materials presenting exclusively Brønsted acidity [44]. In the absence of oxygen, at 180 °C and in the presence of Nb(0.05)-β18 catalyst, glucose is dehydrated to HMF (5-hydroxymethylfurfural) and levulinic acid with selectivities of 22.0% and 8.0%, respectively. However, comparing with the CWO reaction, in the presence of the same catalyst and under 18 atm O_2_ (Table 3, entry 3), the dehydration of glucose takes place with a much lower conversion (47.4%) and after 24 h. Unfortunately, during the glucose dehydration, besides HMF and levulinic acid, a large spectrum of other water soluble side products and unwanted HMF aldol-condensation byproducts, generically called “humins”, are obtained in high amounts. These humans block the active sites of the catalysts, leading to lower catalytic performances. The presence of molecular oxygen seems to be beneficial in avoiding the formation of such unwanted products (i.e., humins). In addition, for all CWO reactions the conversion of glucose is faster and orientated to the targeted reaction product, namely, succinic acid.

However, on Nb-zeolite catalysts the presence of levulinic acid suggests a different reaction pathway to SA compared to Ru-based catalysts [13]. Using Ru-based catalysts only tartaric and fumaric acids have been detected in the reaction products. Under the WO conditions, hydroxy and hydroperoxy radicals are produced from the dissociation and oxidation of water (i.e., H_2_O = •OH + •H; H_2_O + O_2_ = •OH + HO_2_^−^). Hydrogen peroxide can also be formed from the hydroperoxy radicals recombination (i.e., 2HO_2_^−^ = H_2_O_2_ + O_2_) [45]. Then, these radicals together with the free oxygen can attack at the reducing end group, resulting in the opening of the glycosidic ring and the formation of carboxylic acids. In this case, a plausible pathway of the CWO of glucose to the SA product might involve a two-step mechanism: (A) a concerted heterogeneous-homogeneous free radical step leading to tartaric and oxalic acids, followed by the disproportionation of tartaric acid to fumaric and 2,3-dioxosuccinic acids; (B) the catalytic conversion of fumaric acid into SA.

Taking into consideration the literature reports and the experimental results obtained in this study, the formation of succinic acid can also be reasonably explained in the presence of the Nb-zeolite catalysts. According to Ziolek et al. [46] the oxidation of cyclohexene, for instance, with hydrogen peroxide and in the presence of Nb-based catalysts, takes place through superoxo and peroxo species generated through the interaction of NbOH species with hydrogen peroxide:

(2) $$H_{2}O_{2}\;+\;Nb-OH\;=\;HO_{2}^{-}\;+\;Nb-OH_{2}^{+}$$

Once formed, the hydroxyperoxy radicals may interact with hydrogen peroxide hydroxyl with the generation of superoxide radicals:

(3) $$H_{2}O_{2}\;+\;HO_{2}^{-}\;=\;•OH\;+\;O_{2}^{-•}\;+\;H_{2}O$$

The hydroxyl radicals were identified as active species in the formation of cyclohexenediol [47], while the concurrently produced superoxide radicals were considered to be trapped by the amorphous niobium oxide. The reaction equilibrium is in this way efficiently shifted towards the •OH radical formation:

(4) $$O_{2}^{-•}\;+\;Nb(V)\;=\;Nb(V)O_{2}^{-•}$$

**Scheme 1:**
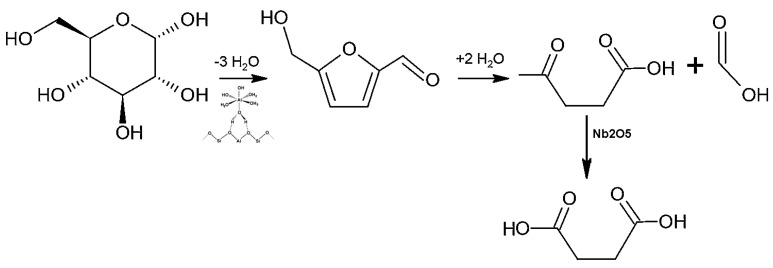
The synthesis of succinic acid (SA) from glucose in two steps.

Similar species can also be formed under the CWO conditions used in this study. Therefore, a two-step mechanism for the production of succinic acid can be proposed, with the synergetic participation of two kinds of active sites: (i) the extra framework AlO_x_(OH) species which dehydrate the glucose to levulinic acid, followed by; (ii) its subsequent oxidation, onto the nano-oxide Nb_2_O_5_ particles, located in the zeolite channels (as extra framework species and/or partly framework penta-coordinated species), in accordance to the above mechanisms (Scheme 1).

These catalysts demonstrated stability under the harsh reaction conditions and can be recycled three times without noticeable variation of the glucose conversion and selectivity to succinic acid (Table 4).

This remarkable stability in water can be due to the presence of the extra-framework aluminum species. When located on the external surface, they prevent the solubilization of the zeolite framework, as recently reported by Sels and co-workers [48].

## 3. Materials and Methods

### 3.1. Catalysts Preparation

Table 5 lists the Beta zeolites subjected to the postsynthetic modification.

#### 3.1.1. Conventional Activation Protocol: Na^+^/H^+^ Ion-Exchange

Except for β6 zeolite, all other zeolite materials were purchased in the NH_4_^+^ form. The ion-exchange protocol was applied only for Na^+^-β6 zeolite, as follows: to a slurry of 1 g of Na^+^-β6 zeolite in water, 100 mL of NH_4_NO_3_ 0.5 M solution was added. The mixture was stirred for 12 h at room temperature. After this time, the solid was separated by filtration, washed with deionized water, dried and subjected to a novel treatment with 100 mL of NH_4_NO_3_ 0.5 M solution. The treatment was repeated four times, yielding a NH_4_^+^-β6 zeolite. The final solid was separated by filtration, washed with deionized water, and dried under vacuum at 80 °C, for 6 h. Finally, the obtained NH_4_^+^-β6 zeolite was calcined in an oven at 450 °C, static air atmosphere, for 10 h, when NH_4_^+^ was decomposed to NH_3_(g) and H^+^, generating the H^+^-β6 zeolite. The calcination temperature was raised with a temperature ramp of 2 °C/min. The same calcination protocol was also applied to all zeolites [49]. The obtained samples were denoted as H-β6, H-β12, H-β18 and H-β37.

#### 3.1.2. Synthesis of Nb-β Zeolites through the Dealumination of β Zeolites and Nb Insertion

Nb-β zeolites (1.6 wt % Nb, 0.02 mol %) were prepared through the two-step post-synthesis method [36]. To obtain a sample with 1.6 wt % Nb, the siliceous (dealuminated) β zeolites were prepared by treating the NH_4_^+^-β zeolite materials (Si/Al = 6, 12, 18 and 37.5) with nitric acid as follows: 2.3 g NH_4_^+^-β zeolite was poured in a solution of HNO_3_ 0.25 N. Then, the mixture was stirred at 80 °C, for 4 h. In parallel, part of NH_4_^+^-β zeolites were treated with oxalic acid 0.1 N using 2.5 g of solid and 50 mL oxalic acid solution. The suspension solution was kept at room temperature, for 8 h [34]. After separation the solid samples were washed with deionized water and dried in an oven at 120 °C overnight. The obtained samples were denoted De-Al-β.

Then, 2 g of the De-Al-β sample were poured in 100 mL of isopropanol solution containing 3.5 × 10^−3^ mol L^−1^ niobium ethoxide (Nb(OC_2_H_5_)_5_) solution and stirred for 3 h, at 80 °C, in an inert atmosphere. Then, the mixture (pH = 5.6) was stirred at 80 °C, for 1 h, in air, until there was a complete removal of isopropanol. The resulted solid was then washed three times with distilled water, dried in air, at 80 °C, for 24 h, and calcined at 450 °C in a static air atmosphere for 10 h [36].

In parallel, similar samples with a final concentration of 0.05 mol % Nb were prepared through similar procedures but adjusting the amounts of raw materials. Table 6 lists all the prepared catalytic samples.

### 3.2. Characterization Methods

The obtained solid catalysts were characterized using powder X-ray diffraction (XRD), diffuse reflectance infrared Fourier transform spectroscopy (DRIFT) and ICP-OES.

Powder X-ray diffraction (XRD) patterns were recorded using a Schimadzu XRD-7000 diffractometer with Cu K_α_ radiation (λ = 1.5418 Å, 40 kV, 40 mA) at a step of 0.2θ and a scanning speed of 2 degrees min^−1^ in the 5–90 degrees 2θ range.

IR diffuse reflectance spectroscopy with Fourier transform (DRIFT) spectra were recorded with a Thermo spectrometer 4700 (400 scans with a resolution of 4 cm^−1^) in the range of 600–4000 cm^−1^.

The contents of niobium and aluminum in the liquid phases were determined by ICP-OES (Agilent Technologies, Santa Clara, CA, USA, 700 Series) after calibrating the instrument with standard solutions.

### 3.3. Catalytic Tests

The activity tests in batch mode were carried out by adding 50 mg of Nb-based zeolite catalyst to a solution of 90 mg (0.5 mmol) glucose in 10 mL of water. After closing, the reactor was pressured at 10–18 bars with molecular oxygen and heated up to 150–180 °C, under stirring (1200 rpm), for 2–24 h. After reaction, the oxygen was released and the catalyst was recovered by centrifugation and the products separated by distillation under vacuum. The recovered products were silylated with 60 μL pyridine, 60 μL BSTFA (*N*,*O*-bis(trimethylsilyl) trifluoroacetamide) and TMCS (trimethylchlorosilane) silane agent, diluted with 1 mL of toluene and analyzed by gas chromatography with flame-ionisation detection (GC-FID) (GC-Shimadzu apparatus, Kyoto, Japan) equipped with a CPSIL8 (commercial name of a GC column with a stationary phase—5% phenyl, 95% dimethylpolysiloxane) column (0.53 mm × 5 μm × 25 m) in following conditions: detector temperature −270 °C; column temperature increased from 45 °C to 250 °C with a ramp rate of 10 °C/min. All samples were analyzed in triplicates. The identification of the products was made by using a GC-MS Carlo Erba Instruments (Waltham, MA, USA) QMD 1000 equipped with a Factor Four VF-5HT column (0.32 mm × 0.1 μm × 15 m).

The glucose conversion (*X*) and selectivities (*S*) to the reaction products were calculated from GC-FID chromatographic analysis by using the follow equations:

(5) $$X\;=\frac{n_{i}\;-\;n{\;}_{t}}{n_{i}}\;\;\times \;100$$

where *n_i_*—initial moles of glucose used in reaction; *n_t_*—moles of untransformed glucose at time “*t*”, determined from gas chromatography (GC) analysis:

(6) $$S_{i}\;=\;\frac{Yield_{i}}{X}\;\times \;100$$

The recovered catalyst was dispersed in distilled water and centrifuged three times, then dried at ambient temperature and used in a consecutive reaction of glucose.

## 4. Conclusions

The insertion of Nb into the Beta zeolite framework is not replacing the tetrahedral positions occupied by aluminum, but following a post synthesis methodology it is possible to afford a very high dispersion of linked Nb(V) species. In accordance to the characterization results, the most probable state of Nb(V) corresponds to Nb(V)O-H where niobium is linked by Nb-OSi bonds to the zeolitic walls. In the preparation process, the tetrahedrally coordinated Al is extracted from the zeolite framework and deposited on the zeolite surface mostly as Al-O extra-framework species. These residual extra-framework aluminum species exhibit a stronger acidity and also prevent the solubilization of the zeolite framework in hot water. Accordingly, bi-functional materials were produced after the post-synthetic insertion of Nb in the zeolite matrix. These new catalysts showed a high efficiency for the one-pot oxidation of glucose to succinic acid. Most probably, the extra-framework aluminum species dehydrate glucose to levulinic acid (LA) which, in turn, is efficiently oxidized to succinic acid (SA) by the Nb species. The novel catalysts can serve as an attractive alternative to the previously developed catalysts based on the Ru critical element. In the presence of Nb(0.05)-β37.5 catalyst, succinic acid is produced in water with a selectivity of 84%, for a total conversion of glucose, after 12 h, at 180 °C and 18 bar of oxygen.

## Figures and Tables

**Table 1:** Crystallinity of the H-Beta sample, calculated with Equation (1).

H-Beta Sample	Si/Al Molar Ratio	Crystalline Phase	Crystallinity (%)
H-β6	6	Beta zeolite	83.1
H-β12	12	Beta zeolite	99.5
H-(β18 and β37)	18 and 37	Beta zeolite	100

**Table 2:** The changes in diffraction line from 2θ 22.48° and the corresponding d_302_ spacing for the Nb-treated catalysts.

Entry	Sample	θ, °	d_302_, Å
1	DeAl-β12 (nitric acid)	22.68	3.917
2	Nb(0.02)-β12	22.57	3.936
3	Nb(0.05)-β12	22.66	3.921
4	DeAl-β18 (nitric acid)	22.67	3.917
5	Nb(0.02)-β18	22.61	3.929
6	Nb(0.05)-β18	22.63	3.926
7	DeAl-β18 (oxalic acid)	22.75	3.906
8	Nb(0.05)-β18O	22.57	3.936
9	DeAl-β37 (oxalic acid)	22.71	3.912
10	Nb(0.05)-β37O	22.38	3.969

**Table 3:** Reaction products distribution in catalytic wet oxidation (CWO) tests.

Entry	Catalyst	X, %	S, %			
			LA	GA	LevA	SA
1	Nb(0.02)-β12	86.2	3.1	5.9	19.0	52.0
2	Nb(0.05)-β12	100	1.0	3.1	5.3	62.3
3	Nb(0.05)-β18	100	3.1	2.1	16.6	72.3
4	Nb(0.05)-β37.5	100	0.6	0.9	3.5	83.6
5 *	Nb(0.05)-β37.5	98.7	2.8	4.3	22.7	58.3
6	Nb(0.05)-β37.5O	100	0	1.3	10.2	70.1

Reaction conditions: 90 mg glucose; 50 mg catalyst; 10 mL water; 18 bar O_2_, 180 °C, 12 h; * −160 °C; LA—lactic acid; GA—glycolic acid, LevA—levulinic acid; SA—succinic acid.

**Table 4:** Activity and selectivity of Nb(0.05)-β18 catalyst in recycling tests.

Entry	Catalyst	X, %	S_SA_, %
1	Fresh catalyst (1st cycle)	86.4	26.1
2 *	After 2 h from the catalyst separation	86.5	26.2
3	2nd cycle	86.0	26.0
4	3rd cycle	86.2	26.1
5	4th cycle	85.8	25.4

Reaction conditions: 90 mg glucose; 50 mg catalyst; 10 mL water; 14 bar O_2_, 180 °C, 6 h; * —the catalyst from entry 1 was separated and the reaction was conducted 3 h longer.

**Table 5:** Commercial Beta zeolites used for the postsynthetic modification.

The Type of the Zeolite Samples	Cation	Si/Al Ratio	The Notation (Symbols)
Beta	Na^+^	6	Na^+^-β6
Beta	NH_4_^+^	12	NH_4_^+^-β12
Beta	NH_4_^+^	18	NH_4_^+^-β18
Beta	NH_4_^+^	37.5	NH_4_^+^-β37

**Table 6:** The prepared catalytic samples.

Zeolite Type	Si/Al Ratio	Dealumination Treatment Acid	Nb Content, mol %	Denoted Nb-Based Materials
Beta	12	Nitric	0.02	Nb(0.02)-β12
Beta	12	Nitric	0.05	Nb(0.05)-β12
Beta	18	Nitric	0.02	Nb(0.02)-β18
Beta	18	Nitric	0.05	Nb(0.05)-β18
Beta	18	Oxalic	0.05	Nb(0.05)-β18O
Beta	37.5	Oxalic	0.05	Nb(0.05)-β37O
